# Topology Effects on Sparse Control of Complex Networks with Laplacian Dynamics

**DOI:** 10.1038/s41598-019-45476-6

**Published:** 2019-06-21

**Authors:** Pedro H. Constantino, Wentao Tang, Prodromos Daoutidis

**Affiliations:** Chemical Engineering and Materials Science Department, University of Minnesota, 421 Washington Ave. SE, Minneapolis, MN 55455-0132 USA

**Keywords:** Chemical engineering, Applied mathematics

## Abstract

Ease of control of complex networks has been assessed extensively in terms of structural controllability and observability, and minimum control energy criteria. Here we adopt a sparsity-promoting feedback control framework for undirected networks with Laplacian dynamics and distinct topological features. The control objective considered is to minimize the effect of disturbance signals, magnitude of control signals and cost of feedback channels. We show that depending on the cost of feedback channels, different complex network structures become the least expensive option to control. Specifically, increased cost of feedback channels favors organized topological complexity such as modularity and centralization. Thus, although sparse and heterogeneous undirected networks may require larger numbers of actuators and sensors for structural controllability, networks with Laplacian dynamics are shown to be easier to control when accounting for the cost of feedback channels.

## Introduction

Large complex networks are ubiquitous in natural and engineered systems, such as gene regulation, metabolic reactions, chemical and energy plants, economics, computer science, social sciences, and many others^1–3^. In the past decade, the modeling of those networks as dynamical systems has motivated the application of control theory to their related problems^4^. Since the seminal paper^5^, considerable research has focused on finding the minimum information needed to control these systems^4–16^. Two concepts that have received a lot of attention are structural controllability and observability^4,5,7^. Given the impracticality of controlling all nodes in a complex network, structural controllability identifies the minimum number of nodes that can guide the entire dynamics of the system and offer full control of the network — commonly referred to as driver nodes^4,5,9,10,12^. Similarly, it is impractical to measure the states of all elements of the network. Structural observability determines the minimum number of nodes that may be monitored in order to infer the dynamical states of all other nodes^4,6–8^.

From a structural controllability and observability perspective, sparse and heterogeneous networks have been deemed difficult to control while dense homogeneous networks have been considered easier^5,12,13^. More specifically, it has been shown for directed networks that clustering and modularity have a modest impact on the minimal number of driver nodes and hence are not advantageous features from a control perspective^12^. The number of driver nodes is mainly determined by the degree distribution and may also be affected by degree correlations^5,12^. From this point of view, it has been argued that real biological networks—such as the ones controlling the dynamics of cellular processes, which are largely characterized by topological features such as sparsity, modularity, hierarchy, and disassortativity—failed to evolve towards structures that are most control efficient^2,4,5^.

The emphasis on structural controllability and observability for control of complex networks, however, neglects the network dynamics and hence provides a limited perspective^6,13,16^. More recent research has considered the dynamics in such complex networks and has proposed different metrics of the difficulty to control them based on “control energy”, i.e. a norm of the control signals that move the state of the network to a desired point in state space^15–28^. Different metrics of controllability have been used to characterize control energy based on the controllability Gramian, including its minimum eigenvalue^15–19^, its trace^20^, the trace of its inverse^21–24^, its condition number^25,26^, and even mixed properties^27^. Some works aimed at the optimal placement of the control nodes to maximize practical controllability of complex networks^20–22,24,28^, while others have focused on relating network structure to the minimum number of control nodes necessary to achieve energetically implementable control profiles^15–19,23,25–27^. The role of modularity (clustering) and centrality in this context has also been evaluated. It has been shown that clustered networks are easier to control based on worst-case control energy^16^ and that removal of edges decreases the control energy and favors increasingly complex network structures in some real biological systems^19^. In fact, isotropic networks^17^ and those in which all nodes have similar centrality^18^, therefore displaying more homogeneous structures, have been shown to be more difficult to control.

In this paper, we study the effect of network topology on difficulty of control in the context of *feedback* control, focusing in particular on the role and significance of *controller sparsity* on this effect. This is motivated by the fact that typical biological networks are regulated via feedback mechanisms rather than open loop control, and that the communication channels between sensors and actuators, i.e. the feedback channels, entail some cost^29,30^. The idea that desirable controllers need to have fewer feedback channels is also common in the theory and industrial practice of automatic control, in the form of decentralized or distributed control^31–35^. Recent work on optimal feedback control costs for complex networks^36^ has investigated performance bounds and the tradeoff between control performance and the number of control inputs for unstable network dynamics.

Here, specifically, we use the sparsity-promoting optimal control design proposed in^37^ as a framework for evaluating control structures in complex networks, and we investigate the network topological features that are most favorable for a broad range of the cost of feedback channels. The formulation used defines a performance cost as the ${ {\mathcal H} }_{2}$ norm of the transfer function from the external disturbance vector to the states and the control input, which relates this performance cost to the closed-loop observability Gramian. In addition, it includes a cost on the number of feedback channels used. It therefore evaluates the capacity of the control system to achieve disturbance attenuation with sparse control.

Our study considers undirected networks with Laplacian node dynamics. The choice of undirected networks is mainly due to the fact that for many molecular and biological networks there is lack of knowledge about directionality and mechanisms of interaction. For example, in protein-protein interaction networks, while nodes representing proteins are known to bind with other molecules, the mutuality of the binding phenomena prevent us from ascribing direction^38,39^. Laplacian dynamics is selected for two main reasons. First, this type of dynamics has been explored at length by graph theorists^40–42^. Secondly, Laplacian dynamics has found diverse applications in physics, engineering, computer science, economics, and lately in biochemical kinetics^40,43–48^. For example, an unexpected relation to stochastic processes and the Chemical Master Equation has been established, which allowed the use of Laplacian dynamics to model gene regulatory networks^43^. The networks analyzed in the paper were artificially generated using the adaptive rewiring method in^49^. Depending on the choice of the network diffusion parameter *τ*, the adaptive method yields networks with different topological features. These networks were subsequently evaluated in terms of minimum achievable performance cost, cost of feedback channels and overall cost.

The results show that for networks with Laplacian dynamics increased cost of feedback channels makes networks with organized topological complexity more advantageous than networks with disordered structures, favoring both modularity and centralization. These results seem to agree with studies reporting that connection costs promote modularity and hierarchy in neural networks^50,51^ and the recent results based on control energy and quantitative metrics of controllability^16–19^. We conclude that despite requiring more driver nodes for structural controllability, from an optimal control cost standpoint sparse and heterogeneous undirected networks with Laplacian dynamics can be easier to control.

## Problem Formulation

We begin with a brief discussion of the network topological features considered in his study. Arguably the most fundamental property of any network topology is its degree distribution^1–3^. Despite the popularity among network theorists of power-law distributions that are parameterized by degree exponents, it is simpler and often sufficient to characterize the degree distribution by the network average degree and sample standard deviation^38,39,52–54^. These may be viewed as estimators of the first and second moments of the degree distribution. The standard deviation of the degree distribution captures the structural effects of the network. For instance, a low standard deviation indicates that the nodes of the network have on average the same connectivity, which also implies that the network may be either random or clustered in multiple modules. Conversely, higher standard deviations indicate that the network is more centralized around particular nodes, i.e. a few nodes form the structural center of the graph and the network develops a star-like topology. Therefore, the standard deviation of the degree distribution is also a measure of network centralization^39^.

Independently of their degree distribution, many networks are structured in such a way that they may be decomposed into sets of communities commonly referred as modules (subunits)^55^. Internally, each module presents many interactions among its components, and performs almost autonomously. Externally, on the other hand, these subunits have few weak interactions among them. Therefore, since these networks only realize a small fraction of all possible interactions among its modules, they are also characterized by sparsity in their connections^38,39,55^. In biological systems, for instance, modules or communities can be identified as patterns of functional and molecular interactions, or also distributions of mutational effects on the phenotype^56–58^. Many biological networks are not only modular but also hierarchical, which means that these modules are also structurally organized^38,50,51^.

As mentioned earlier, for the purpose of this study we generate artificial networks with distinct topological features by tuning the network diffusion parameter (τ) in a recent adaptive rewiring method (*Materials and Methods*, *SI Appendix*). The analysis in the main body of the paper focuses on the degree distribution and modularity effects, which are independent and capture the key trends observed. Results and discussion for other network metrics, such as the average path length, clustering coefficient, assortativity, core-periphery, and PageRank centrality, can be found in the *SI Appendix*.

Laplacian dynamics is considered for the networks generated. The Laplacian matrix is defined as *L* = *D* − *A*, where *D* is the degree matrix, and *A* represents the adjacency matrix, whose elements satisfy *a*_*ij*_ = 1 if there is a connection between nodes *i* and *j*, or *a*_*ij*_ = 0 otherwise. Therefore, this connectivity matrix is symmetric for undirected networks. The state vector ***x*** = [*x*_1_, *x*_2_, …, *x*_*N*_]^*T*^ is governed by the following dynamic equation:

1 $$\frac{d{\boldsymbol{x}}}{dt}=-\,L{\boldsymbol{x}}+{B}_{1}{\boldsymbol{d}}+{B}_{2}{\boldsymbol{u}}$$

where the vector ***u*** is the control input and the vector ***d*** represents the disturbances to the system. We introduce the state feedback control law ***u*** = −*F****x***, where *F* is the feedback gain matrix. We assume for simplicity that all nodes can be manipulated and hence *B*_2_ is the identity matrix. This is also justified by the fact that we are not attempting to solve the problem of minimum inputs to determine the minimum number of control nodes. The dynamic model for the network becomes:

2 $$\frac{d{\boldsymbol{x}}}{dt}=-\,(L+F){\boldsymbol{x}}+{B}_{1}{\boldsymbol{d}}$$

The optimal control problem associated with the networks considered is formulated as in^37^. A control performance-related cost, *J*(*F*), is considered given by the ${ {\mathcal H} }_{2}$ norm of the closed-loop transfer function from the disturbance ***d*** to the output $${\boldsymbol{z}}={[\begin{array}{cc}{\boldsymbol{x}} & {\boldsymbol{u}}\end{array}]}^{{\boldsymbol{T}}}$$:

3 $$J(F)={\rm{trace}}\,({B}_{1}^{T}P(F){B}_{1})$$

Here *P*(*F*) is the closed-loop observability Gramian, which is given by:

4 $$P(F)={\int }_{0}^{\infty }\,{e}^{-{(L+F)}^{T}}(I+{F}^{T}F){e}^{-(L+F)}dt$$

and is obtained by the solution of the following Lyapunov equation:

5 $$-{(L+F)}^{T}P-P(L+F)+(I+{F}^{T}F)=0.$$

Hence, *J*(*F*) measures the impact of disturbances ***d*** on the states and control inputs, which should be minimized. In addition, following^37^, the sparsity of the feedback gain is directly incorporated into the objective function through the definition of a feedback sparsity-related cost as the number of feedback channels, card (*F*), multiplied by the channel cost (γ):

6 $${\rm{minimize}}\,(J(F)+\gamma \,{\rm{card}}\,(F))$$

The minimization of such a total cost gives the optimal controller and the best achievable control cost of the network. We note that even in the case where *F* is not square (i.e. *B*_2_ is not the identity), card (*F*) still refers to the number of nonzero entries of the matrix. For the different networks generated, we compute and compare their control costs, identifying the most favorable network topology under a specific feedback channel cost γ. For details on the solution of this problem please refer to^37^ and the discussion in *Materials and Methods*.

## Results

**Figure 1:**
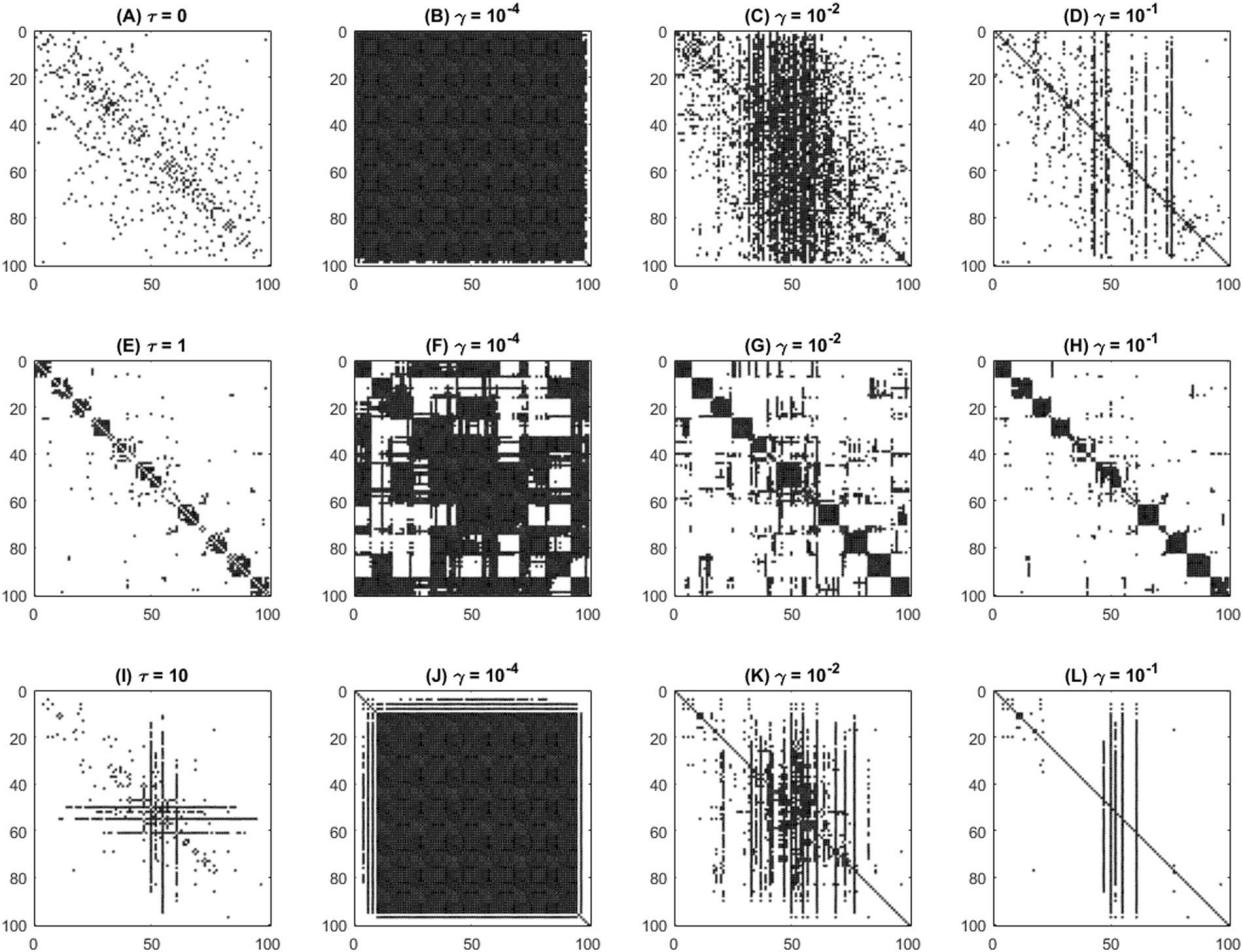
Optimal feedback gain matrix for different network topologies. First column: adjacency matrix displaying network connections. Second to fourth columns: optimal feedback gain matrices with increasing sparsity promotion. First row (**A**–**D**) an ER random network with *Q* = 0.37 and *σ* = 2.48, generated with *τ* = 0. Second row (**E**–**H**) a modular network with *Q* = 0.84 and *σ* = 1.26, generated with *τ* = 1. Third row (**I**–**L**) a centralized star-like network with *Q* = 0.19 and *σ* = 12.26, generated with *τ* = 10. Each nonzero element of the adjacency and feedback gain matrices is visualized as a black dot. Rows and columns of each matrix were permuted to concentrate connections along the main diagonal and improve visualization.

The adjacency (or connectivity) matrix of sampled networks generated with different *τ* values is shown in Fig. 1. When *τ* = 0 the network presents random connections. If *τ* = 1 the network displays more definite community structures laying along the diagonal of the adjacency matrix. Finally, when *τ* = 10 a few nodes of high degree hold most of the connections in a centralized fashion. Figure 1 also shows the optimal feedback gain matrix obtained for three sampled networks within each topology configuration considering different feedback channel costs. When *γ* = 10^−4^ the feedback gain matrix for all network topologies is nearly full and most connections are used since they are cheap, although some sparsity is already observed for modular networks. The minimum total cost among the three networks considered in this case corresponds to the random network. When *γ* = 10^−2^ sparsity increases for all networks but assumes different forms in each topology. Random networks require feedback connections that are seemingly random as well, while modular networks concentrate feedback channels within the communities, and centralized networks favor channels along the star-like center. In this case, the minimum total cost corresponds to the modular network. As the feedback channels become more expensive (*γ* = 10^−1^), they concentrate even further in the communities for modular networks. For centralized networks, however, few high degree nodes dominate the feedback connections and this network topology becomes the option with the sparsest control structure and the minimum overall cost.

**Figure 2:**
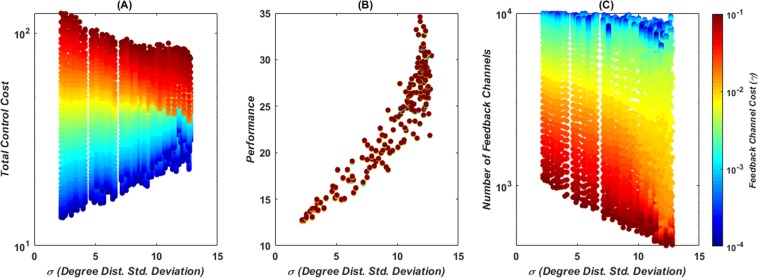
Degree distribution effects on the total control cost (**A**) performance cost (**B**) and number of feedback channels (**C**) from 200 networks with 100 nodes, 300 edges and Laplacian dynamics, generated with the adaptive rewiring method at *τ* = 9 and *τ* = 10. Color map indicates the feedback channels cost.

A more comprehensive description of the effects of degree distribution and modularity of the different networks considered on the optimal cost terms is provided next. The effect of the standard deviation of the degree distribution on the optimal control cost is evaluated in Fig. 2, which shows that the standard deviation has a positive correlation with the control performance cost. When the feedback channel cost is of the order of magnitude of 10^−4^, the optimal number of feedback channels is little affected by the standard deviation of the degree distribution, whereas the total control cost increases with the degree standard deviation. When the feedback channel cost is moderately large, however, the number of feedback channels starts decreasing with increased standard deviation of the degree distribution. The total control cost then becomes largely unaffected by the standard deviation of the degree distribution. When the feedback channel cost is moderate to high both the number of feedback channels and the total control cost decrease with the degree distribution standard deviation. The latter is a reversal of the observed behavior at low costs. This drastic transition becomes visible in the inflection point of the total control cost at large standard deviation values (*SI Appendi*x, Fig. S5).

**Figure 3:**
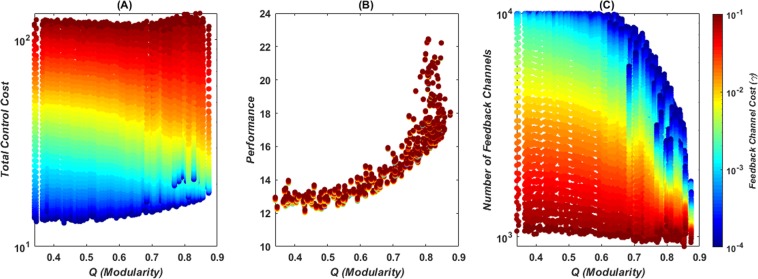
Modularity effects on the total control cost (**A**) performance cost (**B**) and number of feedback channels (**C**) from 400 networks with 100 nodes, 300 edges and Laplacian dynamics. Color map indicates the feedback channels cost. Normalization of the modularity metric to account for variations and structural effects was performed through degree preserving randomizations (*Materials and Methods*). Similar plots for normalized modularity are found in the *SI Appendix* Fig. S7.

Modularity, shown in Fig. 3, also has a positive correlation with the control performance cost. When the feedback channel cost is of the order of magnitude of 10^−4^, increasing modularity decreases the number of feedback channels by nearly 10 times and mildly increases the total control cost. If the feedback channel cost is moderately large, their number starts decreasing with modularity even more strongly than it is observed for the degree distribution. The total control cost also decreases with modularity in this parametric region. Finally, if the cost of the feedback channels is even higher, the optimal number of feedback channels and the total control cost become unaffected by modularity.

**Figure 4:**
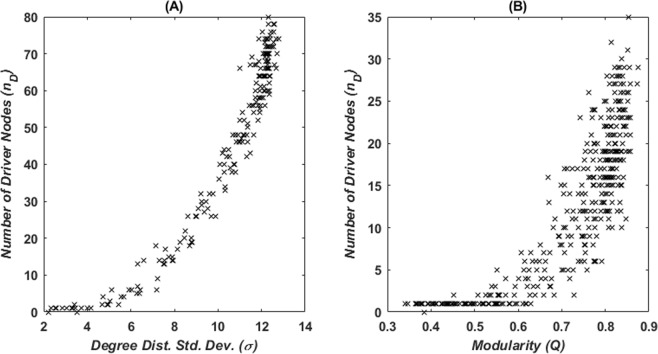
Number of driver nodes versus network metrics. (**A**) Minimum number of driver nodes versus standard deviation of the degree distribution for 200 networks with 100 nodes and 300 edges generated with the adaptive rewiring method at *τ* = 9 and *τ* = 10. (**B**) Minimum number of driver nodes versus modularity for 400 networks generated with the adaptive rewiring method at *τ* = 1, 2, 3 *and* 4. Minimum number of driver nodes calculated from the Popov-Belevitch-Hautus controllability test (*Materials and Methods*).

Figure 4 shows the correlation of modularity and the standard deviation of the degree distribution with the minimum number of driver nodes. We use the method in^9^ for calculating the minimum number of driver nodes for computational convenience although it may result in a smaller estimate due to a different definition of driver nodes used (see also the discussion in *Materials and Methods*). As the standard deviation of the degree distribution grows, the minimum number of driver nodes needed to achieve structural controllability rises from 1 to 80%. A positive correlation, however, is also observed between the minimum number of driver nodes and network modularity after a given threshold value (*Q* ≈ 0.5). The number of driver nodes reaches up to 35% of the network nodes in this case. The structural controllability analysis for the three networks presented in Fig. 1 results in a number of driver nodes of *n*_*D*_ = 1 for the ER random network (Fig. 1, first row), *n*_*D*_ = 12 for the modular network (Fig. 1, second row), and *n*_*D*_ = 72 for the star-like centralized network (Fig. 1, last row). The increased number of driver nodes with larger standard deviations of the degree distribution is consonant with previous research findings for sparse and heterogeneous networks^5,12^. The correlation of modularity with the number of driver nodes, however, to the best of our knowledge, has not been noticed before.

## Discussion

When the feedback channel cost is low, the total control cost is dominated by the performance cost, and the optimal feedback controller is almost centralized, i.e. all the actuators, whether nearby or faraway, are mobilized to attenuate the effects of a disturbance. Compared to networks with modularity or high standard deviation in the degree distribution, a random network has a better capability to propagate the effects of the disturbances beyond the local nodes, and hence disperse the burden of executing control actions on more actuators. Hence, the optimal number of feedback channels shows little correlation with modularity. We conclude that low feedback channel cost favors both non-modular structures as well as networks with small standard deviation from the average degree. Since non-modular networks can only be either random or centralized, we affirm more specifically that low feedback channel cost favors random networks—i.e., homogeneous disordered structures—as the ones that minimize the total control cost.

When the cost of feedback channels is moderate, the decrease in the optimal number of feedback channels with the increase of modularity outweighs the associated increase in control performance cost. Therefore, in this region, modular networks present lower total control cost than non-modular networks do. This, however, holds different for the degree distribution effects. With the increase of the standard deviation of the degree distribution the optimal number of feedback channels decreases in a way that is just enough to compensate the increase in the control performance cost. Hence, the total control cost becomes virtually independent of the standard deviation of the degree distribution. Therefore, our results indicate that moderate feedback channel cost favors modular structures in such a way that may be virtually independent of the network degree distribution.

Lastly, when the feedback channel cost is high, the performance cost becomes less important. In this case, the total control cost behaves similarly to the feedback channel cost. Since the number of feedback channels is unaffected by modularity, the total control cost is also nearly independent of the network modularity. The reason is that for modular networks, the community structures suggest a sparse controller where the feedback channels are mostly concentrated inside the communities^59^, which allows the number of feedback channels to decrease to about 1/*C*, where *C* is the number of communities. When the feedback channel cost is so high that the controller is forced to be “extremely” sparse, the existence of community structures can no longer offer a suitable feedback pattern with such a high sparsity. On the other hand, since the larger standard deviation of the degree distribution decreases the number of feedback channels, the total control cost also decreases when the feedback channel cost is high. In the centralized networks with a degree distribution of high standard deviation, a small portion of nodes has high degrees while most nodes have low degrees. Centralized networks allow such a sparse controller where the cross-node feedback channels are mostly from high-degree nodes to low-degree nodes, so that if the high-degree nodes are disturbed, the low-degree nodes can be informed and mobilized for disturbance attenuation, and otherwise, the nodes can be mostly self-governed. Therefore, our results finally indicate that high feedback channel cost favors networks with degrees distributions that have large deviations from the average node degree. As discussed earlier, this is mainly characteristic of centralized networks with star-like structures.

Several limitations of our approach may be addressed in future work, including the extension to directed networks, networks with different sizes, and networks with other dynamics besides the Laplacian. Future work would additionally include characterizing the transition region from modularity to centralized network structures, which may help determine if hierarchical networks are also favored by the control cost in this formulation. In addition, since the effects of the minimization of actuator, sensors, and feedback channels have been so far investigated separately, they could be considered in a more integrated manner as a single optimization problem. Finally, it would also be of interest to determine whether the minimization of the control cost under sparsity constraints through the feedback channel costs could promote the evolution of networks with modular or centralized features. This would develop into a new network generation method that could shed some light on the possible evolutionary origins of modularity and sparsity in real-world networks.

## Materials and Methods

### Network topology

The modularity metric was defined according to Newman and Girvan^55^. The fundamental idea is that each node of the network must be affiliated to a given community so that the metric may be computed. Modularity is based on local density of connections but since there are several different ways to select module sizes and node affiliation, this task becomes an optimization problem^39,55–57^. The MATLAB Brain Connectivity Toolbox was used for network processing and analysis. This open source toolbox is available at: https://sites.google.com/site/bctnet/. The package contains implementations of the Neumann community finding algorithm for calculating modularity and other common network metrics. Results for other network metrics such as average path length, clustering coefficient, assortativity, core index, and PageRank centrality can be found in the *SI Appendix*.

### Network sampling

We used a recently proposed adaptive rewiring technique in order to generate the networks. The MATLAB code of the algorithm is found in the supplementary information of the open access paper^49^. The method is based on the concept of network propagation, which is increasingly popular among biologists^60^. When *τ* = 0, the time scale of nodal dynamics is infinitely longer than the time scale of rewiring. Then the networks rewire randomly and therefore correspond to Erdős–Rényi (ER) structures. In this parametric region, all network metrics may be considered constant and their distribution provided us with a guideline for what to expect as noise in the sampling process. Starting with a random ER configuration, each network was allowed to rewire 600 times. The probability of random rewiring was fixed at 10%, while the remaining 90% was performed according to the heat kernel diffusion function. During the rewiring process 100 networks were sampled at constant rate. Therefore, we obtained 100 networks for each *τ* ∈ {0, 1, …, 10}, collecting a total of 1,100 networks for processing and analysis. For each sampled network we also generated 1,000 null networks using degree preserving randomizations, i.e. keeping the degree of each node but randomly rewiring all connections in the original network. These null networks were used to normalize the original network metrics and verify if topological features were more abundant than what could be obtained by chance (*SI Appendix*).

We generated a total of 1.1 × 10^6^ synthetic, undirected, simple networks with *N* = 100 nodes and *E* = 300 edges. Therefore, all networks analyzed in this study have the same average degree *k* = 2*E*/*N* = 6. They also present small density *ρ* = 2*E*/(*N*^2^ − *N*) = 0.061, and hence can be described as sparsely connected, which is a general characteristic of many real-world networks such as transcriptional regulatory systems in biology^38,39^.

### Optimal control model

This optimal control problem was solved using the alternating direction method of multipliers (ADMM) following the implementation found in^36^. The open source MATLAB software LQRSP – Sparsity-Promoting Linear Quadratic Regulator and details of the control model are available at http://people.ece.umn.edu/users/mihailo/software/lqrsp/. The state and control performance weight matrices (*Q* and *R*, in the control model) were set to identity for simplicity. Weight matrices for the disturbance vector (*B*_1_) and linear feedback control law (*B*_2_) in the state equation were also set to identity. Therefore, here we consider perhaps the simplest control problem possible. All input options for the optimization model were kept at the default values.

### Driver nodes

The number of driver nodes was determined through the Popov-Belevitch-Hautus controllability test, which extends structural controllability to undirected and unweighted networks^4,9,10^. We have used this method in the absence of other established alternatives for undirected networks and because of its simplicity. A connection between minimum structural controllability (driver nodes) and the PBH theory can be found in^9^. According to the test, the maximum geometric multiplicity of the eigenvalues of the adjacency matrix *A* is equivalent to the minimum number of control inputs, i.e. driver nodes. We note, however, that driver nodes in^9^ appear to be external nodes added to the network rather than existing nodes (states) of the network as is commonly the case. The eigenspace of the network connectivity matrix and the geometric multiplicity was evaluated using the open source MATLAB Teaching Codes toolbox available at http://web.mit.edu/18.06/www/Course-Info/Tcodes.html.

## Supplementary information

Supplementary Information

Dataset 1

## Data Availability

All scripts and graphed data are available in the Supplementary Materials or upon request from the corresponding author.
