# Supplementary material for: Topology Effects on Sparse Control of Complex Networks with Laplacian Dynamics
Source: Sci Rep. 2019 Jun 21;9:9034. doi: 10.1038/s41598-019-45476-6 (PMC6588614; doi:10.1038/s41598-019-45476-6)
Supplement: Supplementary file 1 — Supplementary Information [file 41598_2019_45476_MOESM1_ESM.docx]

Supplementary Information for

**Topology Effects on Sparse Control of Complex Networks with Laplacian Dynamics**

Pedro H Constantino, Wentao Tang and Prodromos Daoutidis

Prodromos Daoutidis

College of Science and Engineering Distinguished Professor

University of Minnesota

Minneapolis, MN 55455

Tel: 612-625-8818

Email: [daout001@umn.edu](mailto:daout001@umn.edu)

**This PDF file includes:**

Supplementary text

Figs. S1 to S7

References for SI reference citations

**Other supplementary materials for this manuscript include the following:**

Datasets S1

Supplementary Information Text

**Other Topology Metrics.** In addition to the degree distribution and the network modularity, there are several other topological metrics which may be used to describe complex networks. Here we briefly mention five of them: clustering coefficient, average path length, assortativity, core-periphery index, and PageRank centrality.

The clustering coefficient considers subsets of nodes sharing many connections as a cluster. It measures the average local clustering, which is the local density of connections (edges) among nodes of the network. If the average clustering coefficient is close to unity, it is likely that the network forms clusters, whereas for random networks the average clustering should approach zero [1-3, 4, 5]. Since, however, this is only an average property, there is also the possibility that a single huge cluster (star-like network) will generate a large average clustering coefficient that fails to imply necessarily the presence of many communities.

The average path length describes the average number of steps that it takes to travel from one node to another when we consider the shortest path between each pair of nodes. When the average path length is small compared to the size of the network, the network is traditionally called small-world [6].

The assortativity coefficient measures degree correlations. More specifically, it quantifies the preference of high degree nodes (hubs) to connect to other high-degree nodes. If the correlation is strongly negative, then hubs tend to avoid interacting among themselves (dis-assortative network). On the other hand, if this correlation is positive, hubs prefer to connect to each other (assortative network). Neutral correlations show more impartiality in the connections, which is characteristic of random graphs [7].

The core-periphery index measures how well we could divide the network into two non-overlapping sets of nodes, namely a core and a periphery. If the index is close to zero, then there is no partition, whereas if the index is close to unity the network is perfectly bipartite [8].

The PageRank centrality sorts the nodes of the network according to their importance based on the eigenvectors of the adjacency matrix and the stationary distribution of a random walk from a given node. Therefore, it is related to the probability of a node being visited in a random Markov chain [9].

**Methods.** Networks generated from distinct diffusion time (τ) values belong to specific distributions of each network metric. We used the Wilcoxon rank sum test to assess the null hypothesis that networks sampled from different diffusion times were from continuous distributions (not necessarily normal) with equal medians. This test can be more easily visualized by the box plot of the network metrics obtained for each τ value. The lower level of each box represents the 25th percentile, while the upper level represents the 75th percentile. The middle red mark displays the median, and the bars extend to the extreme data points not considered outliers, which are in turn represented by discrete points. The notches in each box show the comparison intervals that allow us to confirm if two medians are significantly different. When the intervals from different distributions do not overlap, the test rejects the hypothesis that the samples from each different diffusion time come from distributions with equal medians at the 5% significance level.

For each set of networks obtained from a given parameter τ, we plotted pairwise combinations of network metrics and also modeled a linear regression to investigate the nature of their relationship. In order to quantify more rigorously the statistical significance of the regression we performed a t-test which evaluates the null hypothesis that the slope of the regression was zero at the 0.5% significance level. Failure to reject this null hypothesis combined with the graphical inspection suggested that the metrics were uncorrelated, meaning that variations in one network metric were of little value in explaining the variation in the other. Nonetheless, results from hypothesis testing could be more strict than necessary from a practical perspective due to the relatively large sample size of networks used in this study. Hence, we also considered the looser category of statistical correlations with little or no practical significance when the correlation coefficient between two different network metrics was less than 0.5.

Normalization of the network topology metrics was performed by generating 1,000 degree preserving randomizations for each of them. Following standard approaches from other researchers [10, 11], we used the subsequent equation for normalized modularity:

| $Q_{n}=\left\vert\frac{Q-\left\langle Q_{ran} \right\rangle}{Q_{ran}^{max}-\left\langle Q_{ran} \right\rangle} \right\vert$ | [S1] |
| --- | --- |

Where $Q_{ran}^{max}$ represents the maximal and $\left\langle Q_{ran} \right\rangle$ the average $Q$ measured among the 1,000 degree preserving randomizations. Other metrics were similarly defined.

According to this definition, when $Q_{n}<1$ for a given network, its modularity $Q$ is just an artificial number that could be obtained from any other random network with the same degree distribution. This means that the observed modularity is not a unique feature of the network, but only a structural consequence of its degree distribution. This is commonly referred as structural modularity. On the other hand, when $Q_{n}>1$, then $Q>Q_{ran}^{max}$. Hence, the modularity measured for the original network is more significant than what was obtained for any of the 1,000 random networks.

**Results.** Given that all sampled networks in this study share the same density, they also have the same average degree. Therefore, degree distribution effects are well characterized by the standard deviation. The maximum degree of each network and even alternative graph centralization metrics were tested and yielded similar results, hence being omitted in this report. Our characterization results (Fig. S1) indicate that the range of degree distribution standard deviations from the networks generated with$\tau\leq4$ is narrow and similar to what is obtained for random networks ($\tau=0$). These small $\tau$ values produce networks with wide ranges of average path lengths, mean clustering coefficients, and modularities (Fig. S2). These metrics fail to show any statistical or practical correlation with the degree distribution in this region (Fig. S3). The normalized metrics obtained from degree preserving randomizations show that these network properties still have a wide range when$\tau$ is small (Fig. S4). While the average path length itself displayed no significant correlation with modularity and clustering, the latter were always correlated and consequently generated similar results for the optimal control analysis.

On the other hand, as $\tau$ is further increased, we obtain networks with degree distributions having larger ranges of deviations from the average node degree (Fig. S1). A similar trend is observed for other network metrics such as the assortativity coefficient, the core-periphery statistic and the PageRank centrality (Fig. S2). The linear regression analysis confirms that the standard deviation of the degree distribution for networks generated after $\tau\geq8$ has strong correlations with assortativity, coreness and centrality (Fig. S3).

Additionally, the distributions of the normalized metrics indicate that for all $\tau$ values coreness and centrality have a range that is similar to the range of random networks (Fig. S4). Although the range of centralities may become up to three times larger, the Wilcoxon rank sum test fails to reject the hypotheses that the distributions have the same median. For assortativity, this similar range relative to the random networks is also observed for most $\tau$ values, except between$4\leq\tau\leq7$ (Fig. S4). Moreover, when$\tau\geq9$, even the range of normalized average path length, clustering and modularity becomes considerably small (Fig. S4). While all normalized metrics have decreased ranges when$\tau$ is sufficiently large, only the range of standard deviations from the degree distribution remains large.

**Discussion.** The Wilcoxon rank sum test allows us to assume that the standard deviation of the degree distributions is constant when$\tau\leq4$. Since the degree preserving randomizations for the networks generated within this region were unable to produce similar ranges of average path length, mean clustering coefficient, and modularity, these network properties remain unexplained by the structure of the degree distribution. On the other hand, the distributions of assortativity, coreness and centrality obtained from the degree preserving randomizations were either equivalent to or presented short deviations from the sampled networks. Therefore, the characterization results suggest that the networks obtained from small τ can exhibit various average path length, average clustering, and modularities, while having similar degree distributions with small deviations from the average degree. These networks are more homogenous and virtually neutral in assortativity. Furthermore, any core periphery structure or centrality in these networks is a product of the degree distribution or sheer random noise.

When τ is large all network metrics become less significant than the degree distribution standard deviation. The distributions for normalized coreness and centrality are essentially constant for any $\tau$ value. When$\tau\geq9$, the range of average path length, clustering and modularity are the same as those obtained from the degree preserving randomizations, hence they are a consequence of the degree distribution. All topological features arising more prominently from large $\tau$ values are merely structural and would be observed in most other networks with similar degree distributions. These networks are more heterogeneous and centralized. Therefore, in this parametric region, the degree distribution becomes the main topological feature that characterizes the networks.

In summary, different $\tau$ values allowed us to select networks for which some topological effects are significantly independent from each other. The networks obtained from small $\tau$ values allowed us to investigate how the presence of communities affects the control cost, since the degree distributions and all other structural effects are virtually constant or equally affect all of them as random noise. On the other side of the spectrum, networks obtained from large $\tau$ values allowed us to study the effects of network centralization through the degree distribution independently of other topological features.

Although the network diffusion parameter (τ) and the feedback channels costs (γ) are independent parameters modeling different things, their similar trends in generating or favoring networks with specific topological features is noteworthy. When τ is zero, the adaptive rewiring method produces networks that are random, which are only favored when the feedback channel cost is also close to zero. If we increase τ, we obtain modular networks, which are also the most advantageous to control when we increase the feedback channel costs. Finally, if τ is excessively large, we obtain networks that are centralized and have degree distributions with large standard deviations, which happen to be the favored networks when the feedback channel cost is also large.

This intriguing parallel behavior between the network diffusion parameter and the feedback channels costs leads us to speculate their relationship. This $\tau$ parameter can be interpreted as the ratio of two different time scales, namely the time scale of rewiring and the time scale of network information diffusion. While the time scale of rewiring represents how long the network remains structurally stable, the time scale of information diffusion refers to a single time scale that perhaps simplistically characterizes the entire nodal dynamics of the network. Therefore, we suggest that the nodal dynamics time scale is the main factor dictating the cost of feedback channels. When the time scale for the network dynamics is large (slow dynamics) compared to the rewiring time scale, the network is structurally unstable and the feedback channels cost cheaper. Nevertheless, when the network possesses faster dynamics compared to the rewiring time scale, the network is at a stable quasi-equilibrium structure and the feedback channels become expensive. Further investigation with more detailed modeling of the feedback channels needs to be conducted in order to test these hypotheses.


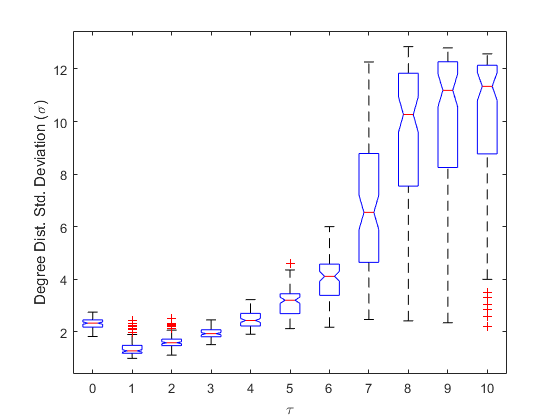


Fig. S1. Box plot of the standard deviations from the degree distribution. For each τ value 100 networks were sampled. Lower level of each box represents the 25th percentile, while the upper level represents the 75th percentile. The middle red mark displays the median, and the bars extend to the extreme data points not considered outliers, which are in turn represented by discrete points.


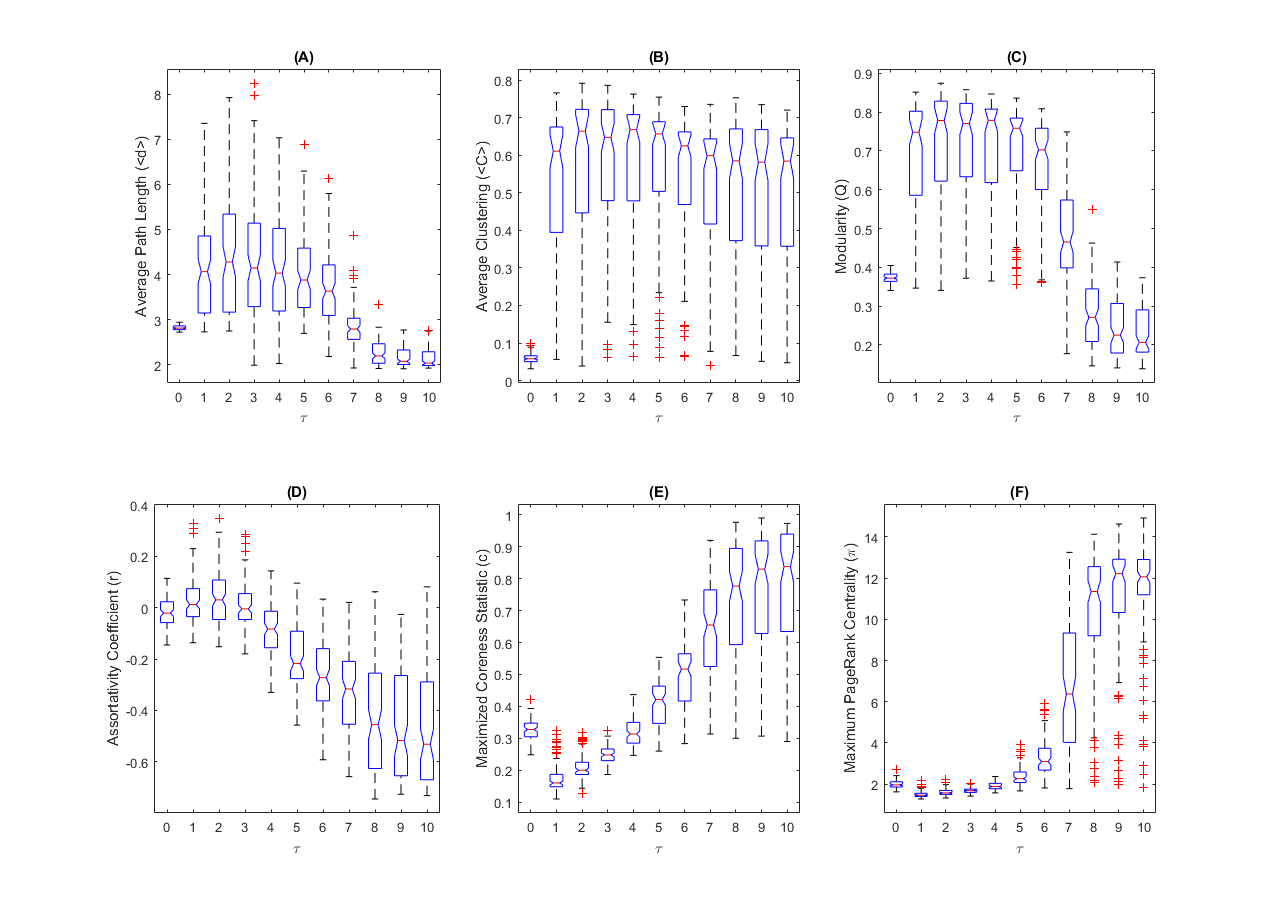


Fig. S2. Box plot of network topology metrics. Distribution of topology metrics for different $\boldsymbol{\tau}$ values. For each value of τ 100 networks were sampled. See details on the legend for Figure S1. (A) Average Path Length; (B) Average Clustering Coefficient; (C) Modularity; (D) Assortativity; (E) Maximized Core-periphery Statistic; (F) Maximum PageRank Centrality.


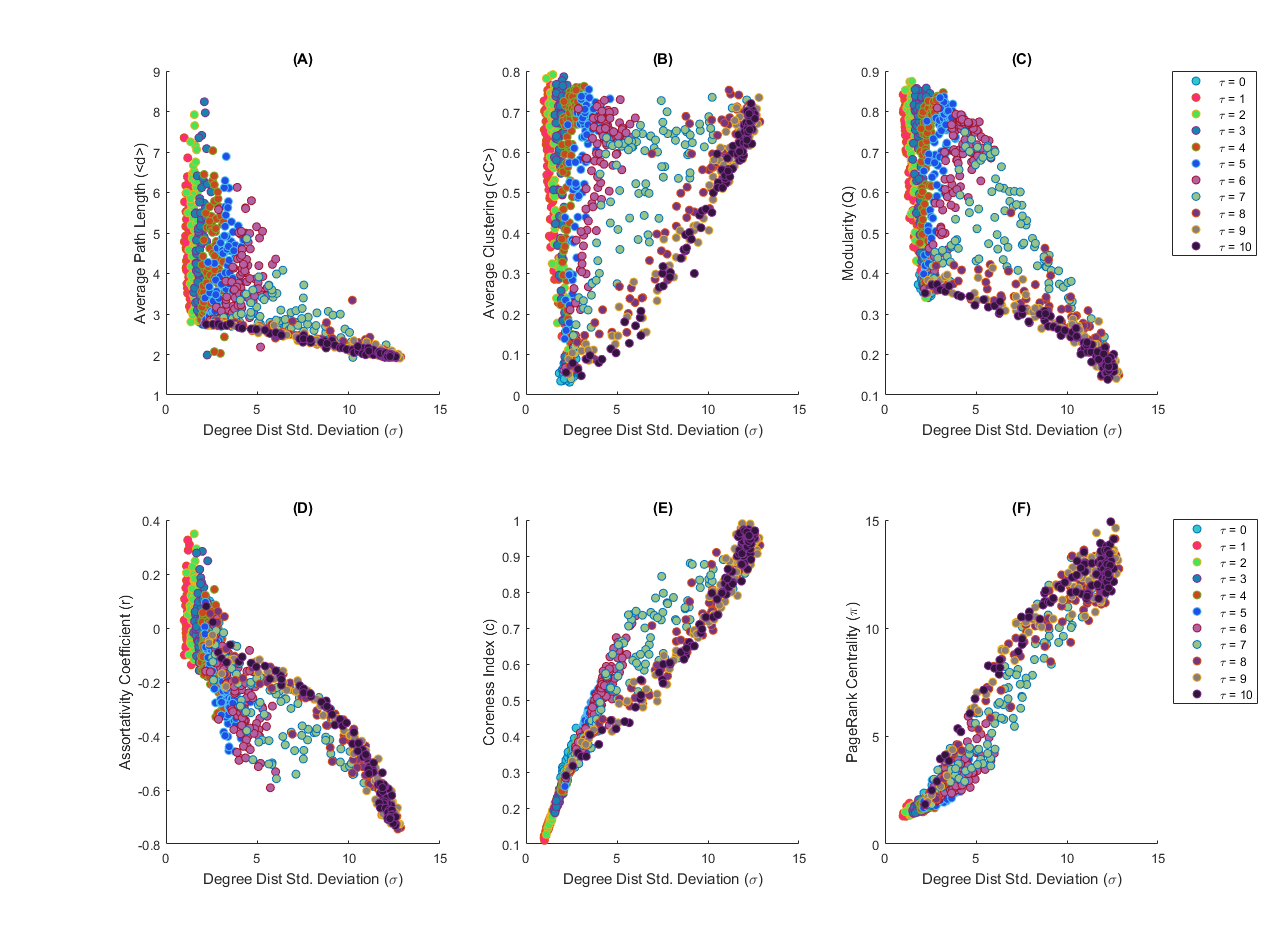


Fig. S3. Correlation between degree distribution and networks topology metrics. For each τ value, represented by a different color (see legend), 100 networks were sampled. Each dot represents a unique network, totalizing 1,100 networks. Presence of correlation between degree distribution and other metrics characterizes inherit structural effects.


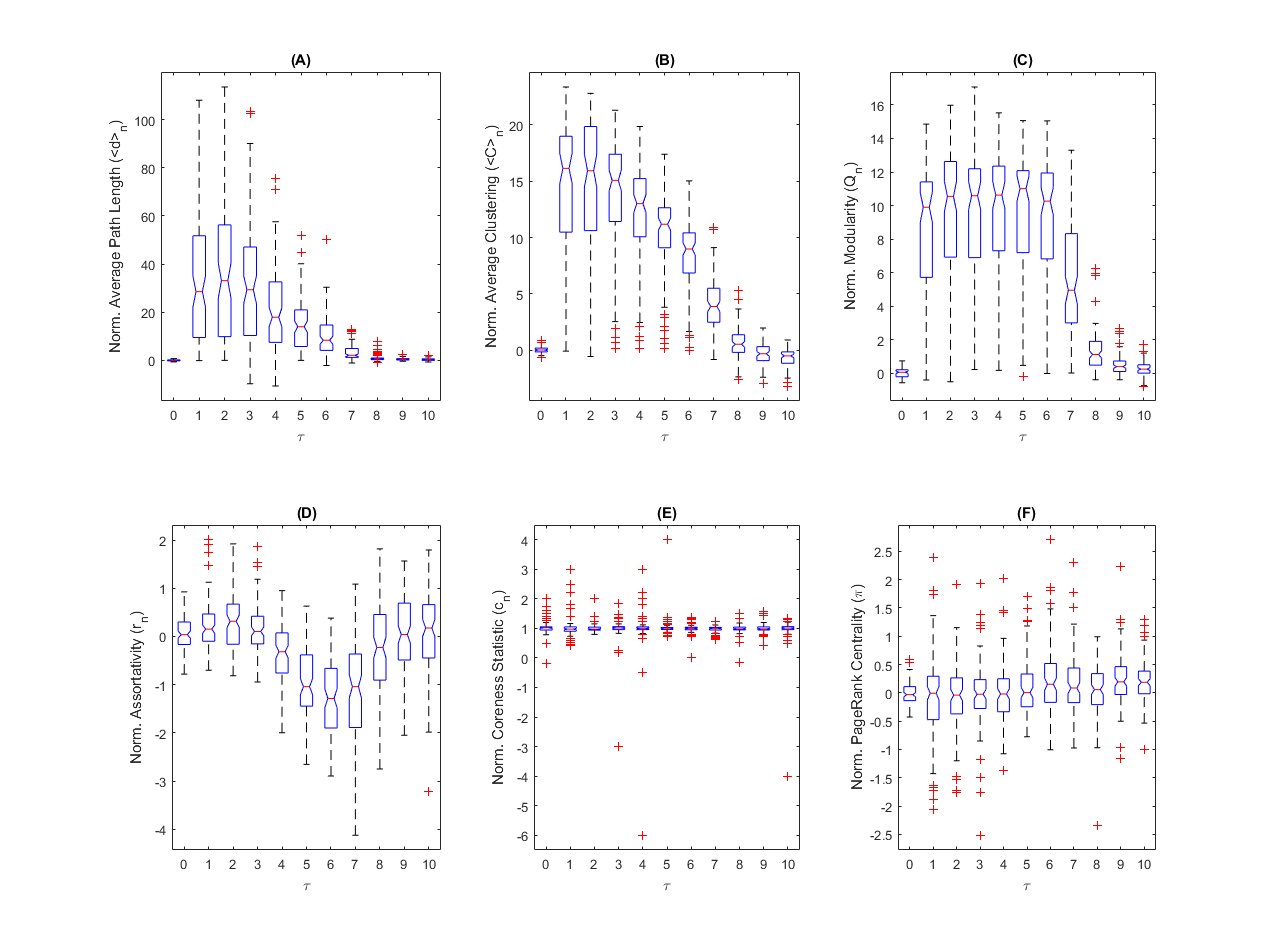


Fig. S4. Box plot of normalized network topology metrics. For each τ value 100 networks were sampled. See details on the legend for Figure S1. For each of these networks 1,000 degree preserving randomizations were generated in order to perform the normalization. (A) Normalized Average Path Length; (B) Normalized Average Clustering Coefficient; (C) Normalized Modularity; (D) Normalized Assortativity; (E) Normalized Maximized Core-periphery Statistic; (F) Normalized Maximum PageRank Centrality.

**
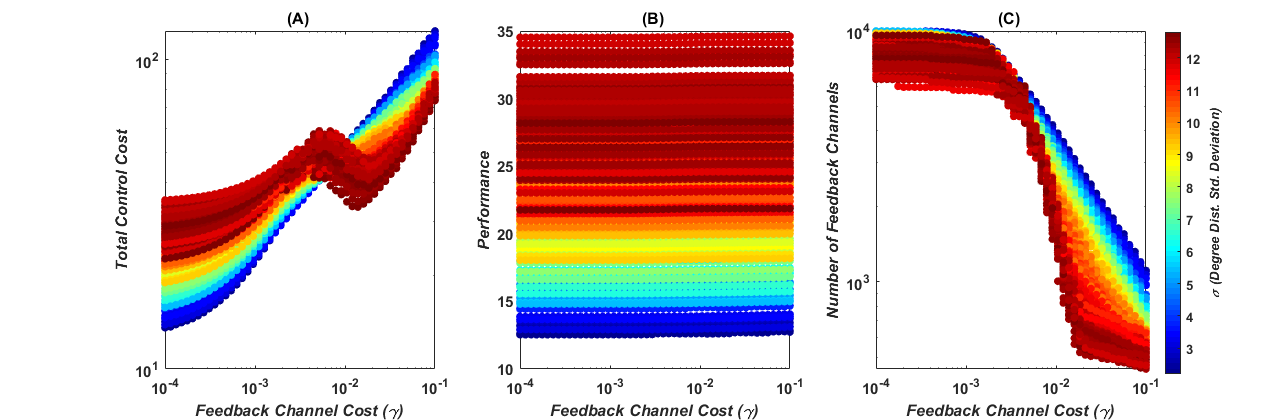
**

Fig. S5. Effects of the feedback channel cost on optimal control for networks with different degree distributions. (A) Total control cost, (B) control performance and (C) number of feedback channels. 200 networks with 100 nodes, 300 edges and Laplacian dynamics. Color map indicates the standard deviation of the degree distribution.

**
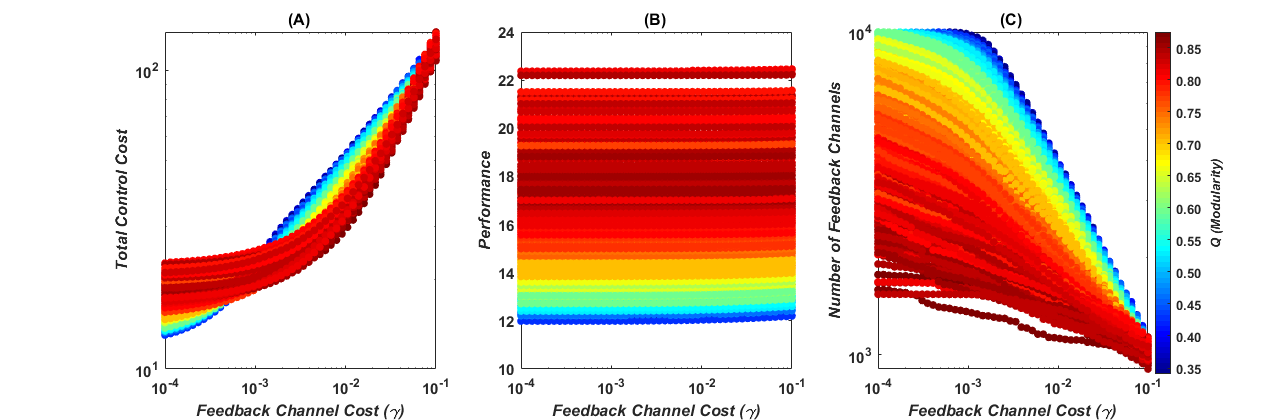
**

Fig. S6. Effects of the feedback channel cost on optimal control for networks with different modularities. (A) Total control cost, (B) control performance and (C) number of feedback channels. 400 networks with 100 nodes, 300 edges and Laplacian dynamics. Color map indicates the network modularity.


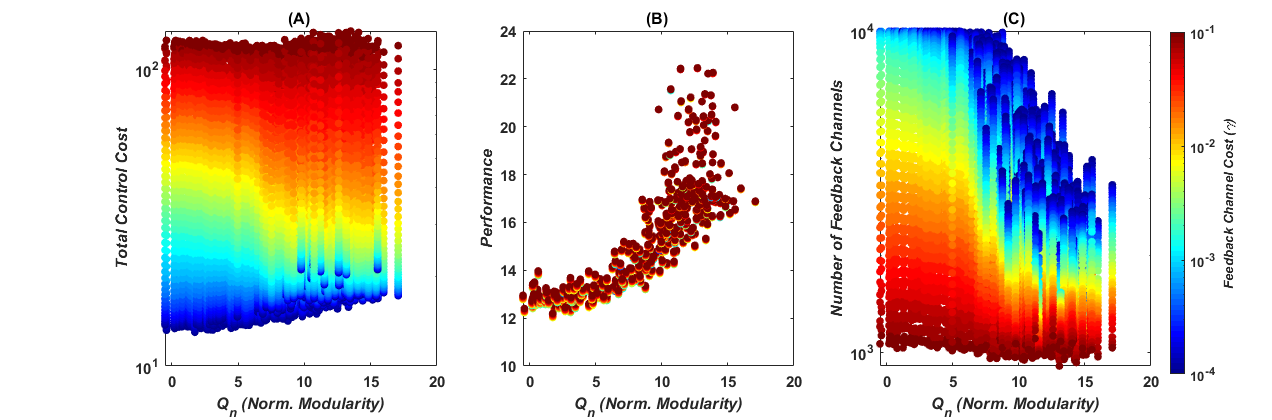


**Fig. S7. Normalized modularity effects** on the total control cost (A), performance (B) and number of feedback channels (C) from 400 networks with 100 nodes, 300 edges and Laplacian dynamics. Color map indicates the feedback channels cost. Normalization of the modularity metric to account for variations and structural effects due to degree distributions was performed through degree preserving randomizations (Materials and Methods).

Additional data (separate file)

**Table S1. Linear Regression Analysis for Network Metrics.** Statistical analysis of the linear regression model $Y=\beta_{0}+\beta_{1}X$ for individual and pairwise correlation between network metrics according to $\tau$ parameters used for network sampling. Data include $R^{2}$ correlation coefficients, t-tests and p-values for the null hypothesis $\beta_{1}=0$. Note that some of these regression models correspond to plots visualized in Fig. S3.

**References**

1. Newman, M. E. J. (2011) Networks: an introduction. Oxford University Press. ISBN-978-0-19-920665-0.
2. Barabási, A., Pósfai, M. (2016) Network Science. First edition, Cambridge University Press. ISBN-978-1-10-707626-6
3. Estrada, E. (2012) The structure of complex networks: theory and applications. Oxford University Press. ISBN-978-0-19-959175-6
4. Pavlopoulos, G. A., Secrier, M., Moschopoulos, C. N., Soldatos, T. G., Kossida, S., Aerts, J., Schneider, R., Bagos, P. G. (2011) Using graph theory to analyze biological networks. BioData Mining, 4:10. DOI: 10.1186/1756-0381-4-10
5. Newman, M. E. J. (2006) Modularity and community structure in networks. Proc. Natl. Acad. Sci. USA 103, 8577-8582. DOI: 10.1073/pnas.0601602103
6. Watts, D. J., Strogatz, S. H. (1998) Collective dynamics of ‘small-world’ networks. Nature 393, 440-442. DOI: 10.1038/30918
7. Newman, M. E. J. (2002) Assortative mixing in networks. Phys Rev Lett 89, 208701. DOI: 10.1103/PhysRevLett.89.208701
8. Borgatti, S. P., Everett, M. G. (2000) Models of core/periphery structures. Soc. Networks 21, 4, 375-395. DOI: 10.1016/S0378-8733(99)00019-2
9. Morrison, J. L., Breitling, R., Higham, D. J., Gilbert, D. R. (2005) GeneRank: Using search engine technology for the analysis of microarray experiments. BMC Bioinformatics, 6:233. DOI: 10.1186/1471-2105-6-233
10. Kashtan, N., Alon U., (2005) Spontaneous evolution of modularity and network motifs. Proc. Natl Acad. Sci. USA, 102, 13 773–13 778. DOI:10.1073/pnas.0503610102
11. Espinosa-Soto, C., Wagner, A. (2010) Specialization can drive the evolution of modularity. PLOS Computational Biology 6(3): e1000719. DOI: 10.1371/journal.pcbi.1000719
